# Recent developments in mass-spectrometry-based targeted proteomics of clinical cancer biomarkers

**DOI:** 10.1186/s12014-024-09452-1

**Published:** 2024-01-30

**Authors:** Deborah Wenk, Charlotte Zuo, Thomas Kislinger, Lusia Sepiashvili

**Affiliations:** 1 Princess Margaret Cancer Centre, University Health Network, Toronto, ON Canada; 2 Department of Medical Biophysics, University of Toronto, Toronto, ON Canada; 3 Department of Paediatric Laboratory Medicine, The Hospital for Sick Children, 555 University Ave, Rm 3606, Toronto, ON M5G 1X8 Canada; 4 Department of Laboratory Medicine & Pathobiology, University of Toronto, Toronto, ON Canada; 5 Sickkids Research Institute, Toronto, ON Canada; 6 Princess Margaret Cancer Research Tower, Room 9-807, 101 College Street, Toronto, ON M5G 1L7 Canada

**Keywords:** Targeted proteomics, Clinical proteomics, Cancer biomarker, Liquid biopsy, LC–MS/MS, Mass spectrometry

## Abstract

Routine measurement of cancer biomarkers is performed for early detection, risk classification, and treatment monitoring, among other applications, and has substantially contributed to better clinical outcomes for patients. However, there remains an unmet need for clinically validated assays of cancer protein biomarkers. Protein tumor markers are of particular interest since proteins carry out the majority of biological processes and thus dynamically reflect changes in cancer pathophysiology. Mass spectrometry-based targeted proteomics is a powerful tool for absolute peptide and protein quantification in biological matrices with numerous advantages that make it attractive for clinical applications in oncology. The use of liquid chromatography-tandem mass spectrometry (LC–MS/MS) based methodologies has allowed laboratories to overcome challenges associated with immunoassays that are more widely used for tumor marker measurements. Yet, clinical implementation of targeted proteomics methodologies has so far been limited to a few cancer markers. This is due to numerous challenges associated with paucity of robust validation studies of new biomarkers and the labor-intensive and operationally complex nature of LC–MS/MS workflows. The purpose of this review is to provide an overview of targeted proteomics applications in cancer, workflows used in targeted proteomics, and requirements for clinical validation and implementation of targeted proteomics assays. We will also discuss advantages and challenges of targeted MS-based proteomics assays for clinical cancer biomarker analysis and highlight some recent developments that will positively contribute to the implementation of this technique into clinical laboratories.

## Background

Cancer is one of the most significant healthcare burdens, with almost 2 million new cases and over 600,000 cancer deaths projected to occur in 2023 in the United States [1]. The cancer death rate has continuously declined since 1991, accumulating in a 33% overall reduction and approximately 3.8 million prevented deaths [1]. This progress can be contributed to advances in treatment, early detection, and risk classification [1, 2]. The routine measurement of cancer biomarkers has played a significant role in these developments.

According to the National Cancer Institute, a biomarker is “a biological molecule found in blood, other body fluids, or tissues that is a sign of a normal or abnormal process, or of a condition or disease” [3]. Biomarkers play crucial roles in cancer diagnosis, monitoring, and determination of disease prognosis, as well as treatment selection [4]. Protein biomarkers are of particular interest since proteins carry out the majority of biological processes [5]. Therefore, their analysis can provide insights into the mechanisms underlying disease and offer a more comprehensive understanding of the clinical condition being studied. Consequently, proteins comprise some of the most frequently requested clinical laboratory tests [6]. Prominent examples of protein cancer biomarkers include prostate-specific antigen (PSA) for prostate cancer screening and early detection of disease recurrence or cancer antigen 125 (CA125) which is used for the surveillance of ovarian cancer recurrence [7–9].

In recent years, liquid biopsies have emerged as a less invasive source for cancer biomarkers than tissue biopsies [4]. The term liquid biopsy refers to the analysis of cancer related signals in bodily fluids, which are assumed to reflect disease-relevant changes in tumor pathophysiology. Common biofluids used for cancer liquid biopsies include blood and urine [4]. Liquid biopsies are often used to investigate cancers proximal to the biofluid. For example, urine has been studied to find biomarkers for cancers of the urogenital system [10, 11], saliva for oral cancers [12], cerebrospinal fluid for brain and CNS tumors [13], pleural fluid for lung cancers [14], ascites for ovarian cancers [15], and stool for colorectal cancers [16]. Unlike tissue biopsies, liquid biopsies permit repeated sampling, which enables longitudinal surveillance and screening for therapeutic resistance during cancer treatment [4].

Currently, immunoassays are predominantly used for the routine quantification of cancer protein biomarkers in the clinic (e.g., PSA, CA125). However, in recent years targeted mass spectrometry (MS) has emerged as a valuable quantitative tool for tumor markers. Despite frequent identification of potentially novel cancer biomarkers in discovery proteomics studies and longstanding clinical use of targeted MS for small molecule quantification, few targeted MS-based proteomic assays have been developed for routine analysis of tumor markers in clinical settings [17] (Table 1). While the reasons are multi-faceted, this can be partly attributed to the paucity of well-designed validation studies demonstrating added clinical benefit and analytical robustness in a manner that meets requirements for analytical and clinical assay validation [7, 18]. Bridging these gaps will substantially increase the translation of newly discovered cancer biomarkers into clinical grade assays. The purpose of this review is to provide an overview of targeted proteomics applications in cancer, workflows used in targeted proteomics, and requirements for clinical validation and implementation of targeted proteomics assays. We will also discuss advantages and challenges of targeted MS-based proteomics assays for clinical cancer biomarker analysis and highlight some recent developments that will positively contribute to the implementation of this technique into clinical laboratories.

**Table 1:** Clinically validated targeted proteomics assays with applications in oncology

Marker	Matrix	Clinical utility	Advantage of targeted LC–MS/MS assay	Methodology of targeted LC–MS/MS assay	Reference
Thyroglobulin	Serum	Monitoring of patients with differentiated thyroid cancers after thyroidectomy and radioactive iodine ablation	Quantification not impacted by anti-thyroglobulin antibodies, which hinder accurate thyroglobulin quantification via immunoassay	Immunoaffinity-based peptide enrichment	Shuford et al. [19]
Chromogranin A	Serum	Monitoring of patients with carcinoid tumors, support diagnosis of carcinoid tumors and other neuroendocrine tumors	Wider dynamic range (quantification over 4 orders of magnitude); increased throughput per batch versus a commercially available immunoassay	Protein enrichment via mixed-mode anion exchange solid-phase extraction	Weber et al. [20]
PTH-related protein	Serum	Diagnosis and management of patients suspected of hypercalcemia of malignancy	Improved analytical sensitivity over commercially available radioimmunoassay; potential for improved diagnosis of PTHrp as a cause of hypercalcemia of malignancy; avoids need to use radioactive tracers used in the established radioimmunoassays	Immunoaffinity-based protein enrichment followed by on-bead digestion	Kushnir and Rockwood [21]
Monoclonal antibody therapies (bevacizumab, evolocumab, nivolumab, pembrolizumab, and trastuzumab)	Serum	Therapeutic drug monitoring, dose optimization	Efficient and reproducible method widely applicable to IgG-based monoclonal antibody therapeutics in plasma	Immunoaffinity-based protein enrichment (IgG) followed by on-bead digestion	Chiu et al. [22]
Rituximab	Serum	Therapeutic drug monitoring	Precise and accurate method with high inter-lab concordance	Immunoaffinity-based protein enrichment (IgG) or trichloroacetic acid/isopropanol-based albumin depletion	Millet et al. [23]
Cetuximab	Serum	Therapeutic drug monitoring	Quantification not impacted by endogenous interferences that may impact ELISA measurements	High pH peptide fractionation No protein or peptide enrichment/fractionation	Becher et al. [24] Shibata et al. [25]

## Targeted proteomics: definition and workflows

MS is a powerful analytical technique that determines the mass of molecules after ionization. Targeted proteomics applications enable absolute quantification by utilizing tandem mass spectrometry (MS/MS), where two mass spectrometers are coupled to each other via a collision cell: The first mass analyzer selects intact molecule ions (precursor ions), which are afterwards funneled into the collision cell to be broken down into pieces (fragment ions). The second mass analyzer then detects these fragment ions. Pairs of precursor and fragment ions are referred to as ion transitions. For increased specificity, minimum two transitions are monitored for a single analyte with one of the most selective transitions being used for quantification (“quantifier ion”) and another to verify the identity of the analyte (“qualifier ion”) [26]. MS/MS can be used both for screening of a sample without prior knowledge of the targets (untargeted MS) as well as for quantification of preselected analytes (targeted MS). Untargeted MS is a valuable tool for the discovery of novel biomarker candidates, while targeted MS is better suited for biomarker assay development and validation due to its higher sensitivity, accuracy, and precision compared to untargeted MS.

**Fig. 1:**
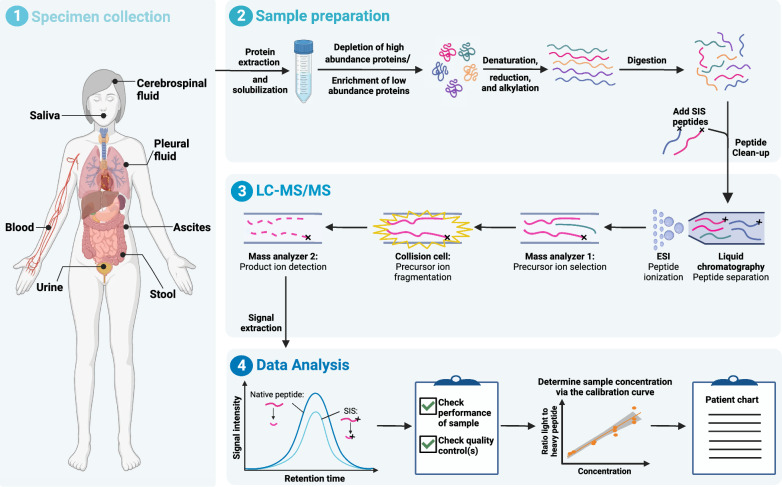
Typical workflow for the analysis of protein cancer biomarkers from liquid biopsies via targeted mass spectrometry. *SIS* stable isotope labeled standard, *LC* liquid chromatography, *MS/MS* tandem mass spectrometry, *ESI* electrospray ionization. Figure created with Biorender.com

In a typical workflow for the quantification of protein biomarkers from liquid biopsies via targeted MS, proteins are digested to peptides before their separation via liquid chromatography (LC) and analysis via MS/MS (Fig. 1). A protein or peptide depletion or enrichment step can be added to remove high abundance proteins from the sample or enrich for target analytes, which improves the detection of the lesser abundant cancer biomarkers. This is especially needed for the analysis of serum or plasma, since the 22 most abundant proteins make up 99% of the serum protein mass [27], but is also routinely applied to other sample types such as urine [28] or cerebrospinal fluid (CSF) [29].

For quantification of biomarkers, their signal is compared to stable isotope labeled standards (SIS) that are added to each sample during sample preparation. SIS are compounds in which several atoms in the analyte are replaced by their stable isotopes, such as 2H (D, deuterium), 13C, 15N, or 17/18O [30, 31]. They have the same (or highly similar) physicochemical properties than their endogenous (light) counterpart which means that they show the same behaviour during sample preparation, LC separation, and electrospray ionization (ESI), but they can be distinguished in MS due to their larger molecular mass. Since targeted proteomics workflows measure peptides that result from a (usually tryptic) digest of a sample, they most often utilize SIS peptides that are added after the digestion step (Fig. 1). However, to account for analytical variations prior to and including digestion, the use of SIS proteins or winged peptides (where the tryptic peptide sequence is extended at the C- and/or N-terminus by a few amino acids) is recommended, albeit this is not always feasible and more expensive [32].

## Regulatory requirements for clinical targeted proteomics assays

**Fig. 2:**
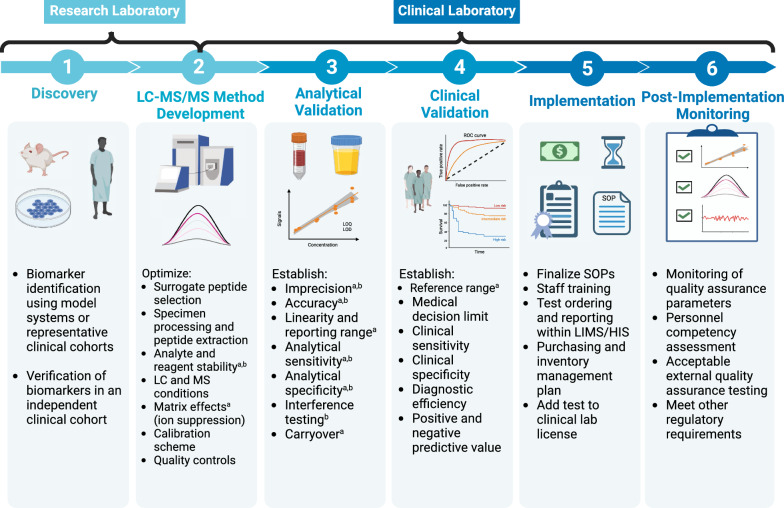
The path to clinical implementation of a targeted LC–MS/MS assay. Targeted LC–MS/MS assays for tumor markers may be developed following identification and verification of novel tumors markers in the research setting, alternatively they may be developed for established markers due to limitations or lack of alternative methodologies (Panel 1). This path may not be as linear as shown due to challenges that may be encountered at the various stages necessitating a return to the method optimization stage, method validation, or updates in post-implementation monitoring protocols. ^a^Denote analytical performance metrics that require validation for laboratory-developed tests as per the College of American Pathologists. ^b^Prior to investing significant resources in a method validation, pre-validation evaluation may be performed to assess key parameters. The extent of clinical validation may vary and may involve establishing a reference range based on analysis of an apparently healthy cohort [37], verifying a reported clinical cut-off or medical decision limit (CLSI), or establishing a clinical cut-off [38]. *LIMS* Laboratory Information Management System, *HIS* Health Information System. Figure created with Biorender.com

In order to translate a targeted proteomics assay into clinical practice, numerous key steps should be undertaken with the goals to establish and meet clinical and analytical performance metrics while being compliant with rigorous regulatory requirements for test validation and ongoing test performance (see Fig. 2). Clinical laboratory regulations stipulate requirements for facility administration, quality management systems (quality assurance and quality control), external quality assurance, personnel, and laboratory inspection; these should be met for all clinical laboratory tests irrespective of analytical methodology (see Lynch, K.L. for a more detailed description [33]).

There are currently no FDA-approved targeted proteomics LC–MS/MS assays. Therefore, targeted proteomics assays fall in the category of “Laboratory Developed Tests” (LDTs) and necessitate method development, validation, and implementation by laboratories certified and accredited to perform clinical lab testing on the intended instrumentation. A LDT is defined by the FDA as a test that is designed, manufactured, and used within a single clinical laboratory [34]. As the first step, prior to beginning any method development activities, a feasibility assessment is conducted which involves detailed justification of the clinical benefit of the assay and its intended use, defining test characteristics, and outlining regulatory and practical considerations and risks [35]. Feasibility assessment typically involves extensive consultation between clinical and laboratory stakeholders. The gathered information serves as the basis for defining goals for analytical and clinical performance of the method which should be met following method validation. Analytical performance metrics include goals for accuracy, imprecision, analytical sensitivity, the range of concentrations the assay should be able to measure (reporting range), among others. As the next step, method development and optimization can begin, followed by method validation once the entire analytical workflow has been defined. Key performance characteristics for method development and validation are highlighted in Fig. 2**.** Numerous documents published by the Clinical & Laboratory Standards Institute (CLSI) provide detailed guidance on test development and validation with “C50-A: Mass Spectrometry in the Clinical Laboratory: General Principles and Guidance” [36], “C62-A: Liquid Chromatography-Mass Spectrometry Methods” [26], and “C64 Quantitative Measurement of Proteins and Peptides by Mass Spectrometry” [35] of particular relevance to targeted proteomics methods.

Once validation is complete and pre-defined analytical and performance requirements are met, there is an extensive implementation phase involving many members of the clinical laboratory including laboratory director, laboratory management, medical laboratory technologists performing testing, quality management team that supports regulatory compliance, information technology team supporting LIS/HIS test integration, and medical laboratory technicians and clinical staff who may be involved in specimen collection and handling, and others. Therefore, implementation requires careful coordination and excellent communication.

Post-implementation, a quality assurance program ensures patient results can be reported reliably and minimizes risk of erroneous results. This includes internal quality assurance, quality control, and external quality assessment (Fig. 2 and Table 2). The acceptance criteria for internal quality assurance and quality control parameters are typically established during method validation; runs or specific samples are rejected and cannot be reported to the patient chart if these criteria are not met. External quality assurance involves periodic blinded testing of specimens and comparison of obtained values with other laboratories or target values obtained via a reference method through a third-party proficiency testing program [17, 33]. In cases such programs are unavailable (e.g. in case of novel protein cancer biomarkers), alternate approaches such as split sampling and comparison of values with another clinical laboratory, or another clinically available method should be employed. The lab director is responsible for establishing these alternate approaches and review all proficiency testing results. Discordant results require investigation, analysis of impact on patient results, and implementation of a corrective action plan if necessary.

**Table 2:** Quality assurance of LC–MS/MS assays [17, 26, 33, 35, 36]

System suitability assessment (SSA): System suitability material (e.g. mixture of internal standards and/or target analytes in pure solvent) is analyzed following system equilibration and priming to ensure acceptable LC–MS system performance prior to commencement of an analytical run. SSA acceptance criteria established during method validation should be met for pre-defined chromatography metrics such as peaks eluting at expected retention times, adequate analyte and background signal, etc
Calibrator accuracy and calibration curve metrics: Each point in the calibration curve should meet pre-specified allowable bias metrics (e.g. ± 15% of target value). Additional calibration curve metrics include the slope, and r^2^ supporting the linear response between analyte response (peak area) to that of SIS (y-axis) and known analyte concentrations (x-axis). The metrics should be defined based on variation observed during method validation and informed by clinical guidelines, and regulatory requirements
Quality control monitoring: Quality control samples containing known analyte concentrations should be included throughout the batch and yield concentrations within an established range (e.g. mean ± 2 standard deviations). The ranges are established for each lot and level of quality control through repeat analysis within and between multiple runs. Runs are rejected in case of unacceptable quality control. Quality control samples should span clinically relevant concentrations
Internal standard monitoring: Ensures internal standard peak area for each sample is within acceptable recovery limits established during method development. The SIS peak area should be within acceptable limits for calibrators, quality control and patient samples. If outside limits, specimens are to undergo repeat analysis and be rejected from being reported to the patient chart if this does not resolve the issue
Ion ratio monitoring: Ion ratios, the ratios of the peak area of the qualifying ion transitions to the peak area of the quantifying ion transitions, should be reviewed for each patient sample and IS. Patient samples with ion ratios falling outside of of predefined limits should undergo troubleshooting as they may have interferences preventing reliable quantification. The criteria for the predefined limits are determined during method development, prior to method validation
Retention time monitoring: Analyte and SIS peaks are checked for chromatographic retention times during and between analytical runs to ensure they are constant and within acceptable tolerance limits. Retention times between analyte and IS in patient samples and QC should be similar to the standards
Lot change evaluation: In order to ensure consistency of patient results and avoid introduction of significant analytical biases, prior to implementation of new lots, patient samples (n ≥ 5) should be analyzed on new and current lots of critical reagents and consumables and values should compare within a pre-specified allowable bias limit. Critical reagents include new lots of calibrators, internal standards, calibrator matrix, and any other reagents that may impact analytical performance. Consumables include LC columns

## Advantages of targeted mass spectrometry

### Selectivity and specificity

One of the key advantages of targeted MS is its high selectivity and specificity. In contrast to immunoassays, which rely on the interaction of an analyte with an antibody, MS/MS directly measures a target and confirms its identity via its fragments. MS is therefore less susceptible to interferences commonly encountered by immunoassays which can contribute to diagnostic error. Immunoassays can be impeded, for example, by interferences via cross-reactivity, presence of autoantibodies against the target protein, heterophile antibodies, other anti-reagent antibodies, or non-specific binding [39, 40]. Consequently, targeted MS has been proposed as an alternative to immunoassays that are hindered by such interferences. For example, serum thyroglobulin measurements via LC–MS/MS are resistant to interference by anti-thyroglobulin antibodies that hinder its quantification by commercially available immunoassays in approximately 25% of differentiated thyroid cancer patients [39]. However, LC–MS/MS-based assays might have other drawbacks that hinder them from becoming the standard method for single markers such as their labor-intensive nature and analytical complexity.

### Applicability to different specimen matrices

MS-based proteomics workflows commonly include protein/peptide extraction and cleanup steps, this in combination with MS’s high selectivity means that targeted MS assays are adaptable to diverse sample matrices. For example, Chi et al. investigated 30 potential oral cancer biomarkers in matched serum and saliva samples from 30 oral cancer patients and 30 healthy controls with the same targeted proteomics assay, finding five candidate biomarkers with disease-discriminating powers (AUC > 0.75) in saliva, but none in serum [41]. In another study, Adrait et al. utilized targeted MS to study cancer biomarkers in bile, a matrix that is rarely validated for immuno-based assays [42].

### Multiplexing capabilities

Targeted MS assays can precisely quantify hundreds of analytes [2], as shown for example in large-scale cancer biomarker studies by You et al. [43] or Sinha et al. [44]. To maintain analytical sensitivity and accuracy, targeted MS methods for a large number of targets are often refined to scheduled PRM/MRM methods, where analytes are only monitored in a limited time window around their expected retention time. This ensures that the MS method can still gather enough data points across each chromatographic peak for accurate quantification by limiting the number of concurrently monitored precursors. Assays with multiplexing capabilities are especially useful for monitoring biomarker panels. Marker panels have the potential to be more clinically sensitive and specific than single biomarkers and can thus have higher diagnostic efficiency or predictive value [45–50]. Additionally, biomarker panels can help to classify tumors into subtypes that can be used to guide personalized treatment decisions [51]. Immunoassay platforms’ multiplexing capabilities have also been further developed in recent years (for a review see Ren et al. [40]). However, the highly multiplexed immuno-based platforms (e.g., Olink, SOMAscan) are often not validated for routine clinical analysis and suffer from specificity issues and low inter- and intra-assay reproducibility [52, 53]. For example, Joshi et al. observed that 7% of SOMAscan aptamers bound to different proteins and that 32% of aptamers displayed altered binding affinity due to Single Nucleotide Polymorphisms [52].

### Sample efficiency

Targeted MS assays can quantify numerous analytes using small sample volumes due to their multiplexing capabilities. Targeted proteomics studies have used sample amounts as low as 500 μl of urine [48], 25–50 μl of serum [44, 54], or even dried blood spots [55] to quantify hundreds of proteins. The low volume requirement is suitable for pediatric applications, and LC–MS/MS methods are routinely applied in newborn screening programmes that test for congenital disorders via metabolic profiling [17].

### Dynamic range

Biological samples are usually complex in nature with a wide dynamic range of protein concentrations. Workflows combining specimen extraction (including enrichment or depletion) and liquid chromatography separation of peptides help overcome the limitations of low abundance protein quantification in complex biological matrices. Targeted MS assays can quantify precisely over 5 to 6 orders of magnitude [40, 56], while most immunoassays in clinical use have a dynamic range of 3 to 4 orders of magnitude [40]. Accordingly, MS is often considered as the method with the larger dynamic range. Newer immunoassay platforms based on proximity extension (Olink) or slow off-rate modified aptamers (SOMAscan) can achieve dynamic ranges of up to 10 orders of magnitude through the use of serial dilutions [40], however those technologies are often not validated or available for clinical use (see *Multiplexing capabilities*).

### High reproducibility

Targeted LC–MS/MS methods deliver highly reproducible results, including when different laboratories employ an assay [57]. This holds true even when different enrichment and calibration protocols are being used [58] or the MS-based assay targets different peptides [59]. This is largely due to the utilization of internal standards that correct for any analyte losses during sample preparation and matrix effects during analysis [26]. Immunoassays, on the other hand, suffer from larger long-term and inter-assay variation, due to lot variability [60] and the fact that antibodies used in immunoassays from different manufacturers usually target different epitopes [39]. This can result in discrepant results and have significant impacts on clinical decision making: test results by one laboratory might indicate a therapy need while results by another laboratory do not or a patient might be misdiagnosed with recurrent cancer when transferring to a new hospital [61].

### Transition from discovery to application

Untargeted LC–MS/MS methods used for discovery of biomarker candidates can be adapted relatively quickly to targeted LC–MS/MS assays and thus further speed up the discovery-to-application process [62]. Firstly, the proteomics sample preparation used in the discovery stage can be easily adapted for the targeted LC–MS assay since workflows used for untargeted and targeted LC–MS-based proteomics contain the same basic steps (Fig. 1). Substantial differences include that targeted proteomics assays often use protein depletion or enrichment instead of more extensive protein/peptide fractionation and that assays used for absolute quantitation need to incorporate SIS proteins or peptides. Secondly, the performance of candidates in untargeted proteomics can be used to prioritize biomarker candidates for the targeted assay due to the similarity of the two approaches. Thirdly, SIS peptides needed for targeted assays can be synthesized relatively quickly (usually within a few months). In contrast, clinical development of an immunoassay in the absence of a commercially available kit or antibody is impractical since the large-scale production and characterization of high-quality monoclonal antibodies for immunoassays are labour-intensive, time consuming (years), and cost-prohibitive [63].

### Versatility

LC–MS/MS can be used to quantify not only proteins, but also small molecules such as metabolites, hormones, drugs, and environmental contaminants, as well as carbohydrates, nucleic acids, and lipids [17]. This versatility allows clinical laboratories to employ one MS system for several applications and can compensate for the relatively high acquisition and maintenance costs of MS systems (see next section).

## Challenges of targeted mass spectrometry

### High technical proficiency

Mass spectrometers and mass spectrometry methods are relatively complicated to use, requiring expert knowledge that is usually not taught to personnel certified to perform clinical testing [17]. Required expertise includes MS calibration, LC column equilibration and testing, MS and LC troubleshooting, setting up LC and MS methods, and data analysis, amongst many other things. Immunoassays, in contrast, are more familiar to medical technologists as immunoassay analytical principles and hands on exposure are a standard part of medical technology programs. Additionally, commercially available immunoassays are equipped with a detailed standard operating procedure (SOP), can sometimes be placed on fully automated platforms, and have fewer parameters requiring optimization during method development. Consequently, MS systems need to become easier to use to make the technology more accessible to clinical laboratories.

### High cost

Mass spectrometers can cost up to 500,000 USD or more [64], making LC–MS/MS equipment generally 3 to 6 fold more expensive than immunoassay instruments [39]. Additionally, preparation of reagents and samples for MS-based protein analysis is more extensive and requires more hands-on time than immunoassay workflows, increasing the amount of technologist time (and thereby labor cost) per sample [39]. Lastly, the high technical proficiency needed for maintaining and troubleshooting MS systems require to either hire or train a MS specialist and/or to sign a service contract with the MS instrument vendor, which further increases costs [17]. However, since LC–MS/MS can be used for many different applications including metabolite screening, therapeutic drug monitoring and others [17], the acquisition of a LC–MS/MS system can be cost-effective for laboratories with a diverse test menu. Additionally, the higher specificity of MS-based assays can decrease the healthcare costs associated with the management of patients. For example, MS-based assays for serum thyroglobulin measurement are resistant to interference by anti-thyroglobulin antibodies, circumventing the need for long-term follow-up of differentiated thyroid cancer via costly methods such as neck ultrasound, radioiodine diagnostic whole-body scans, or fluorodeoxyglucose-positron emission tomography imaging [39].

### Manual sample preparation

The preparation of proteomic samples for LC–MS/MS analysis (Fig. 1) is often performed manually, especially in research settings. This reduces throughput and increases analytical imprecision. Especially the digestion step is a major source for variability in LC–MS analysis [65]. The proteome’s high complexity (> 20,000 genes, multiple isoforms, and post-translational modifications) [66] and the large dynamic range of protein concentrations complicate proteolytic cleavage reactions in different matrices [65]. Thus, careful optimization of the proteolysis conditions (as well as denaturation, reduction and alkylation, and peptide clean-up), can be necessary to achieve inter‐ and intra‐day imprecision of < 20% [65, 67]. However, most developed workflows for LC–MS/MS based targeted proteomics are highly variable between laboratories due to the fact that the majority of LC–MS/MS-based assays in the clinical setting are LDTs [33]. They often lack traceable certified reference materials and commercially available quality controls, calibrators, reagent kits, as well as published SOPs from sample preparation to data processing [17, 68]. These limitations need to be overcome to standardize LC–MS/MS based proteomics assays and thereby make the technology more accessible and positively impact data quality and, consequently, patient safety [17]. Additionally, available automated sample processing platforms should be implemented to improve the throughput and further reduce the variability of LC–MS/MS based proteomics assays.

### Turnaround time

A test’s intra-laboratory turnaround time can be calculated as the time from specimen collection to the time the result is reported and populates a patient chart. Short turnaround times (minutes or hours at most) are required for clinical assays used to facilitate clinical decision making in emergency and critical care settings; in outpatient settings longer turnaround times may be acceptable (e.g. several days). Therefore, depending on the intended use, consideration may need to be given to minimizing turnaround time and improving efficiency when translating a cancer biomarker or panel from research to the clinic. LC–MS/MS based targeted proteomics workflows typically have turnaround times of several days, whereas most automated immunoassays take less than 30 min per test and can produce same-day results [6]. Crucial steps that influence the turnaround time of proteomics workflows include proteolysis [65] (commonly 4 to 18 h), peptide clean-up, and LC–MS/MS measurement. Each sample, quality control, and calibration solution are measured individually via LC–MS/MS, consequently short methods are key to achieving high throughput and thereby quick turnaround times for large numbers of samples. For example, the typical routine clinical LC–MS/MS analysis time for small molecules is 2–5 min [17, 26]. In contrast, many LC–MS/MS methods used for untargeted proteomics in discovery studies take 2 to 4 h.

### Sensitivity

LC–MS/MS is susceptible to ion suppression, a phenomenon where the presence of endogenous or exogenous compounds during the ionization process can alter the ionization efficiency of the target analyte and thereby compromise precision and sensitivity of the assay [69]. Ion suppression is particularly apparent in complex samples such as biofluids, and can lead to immunoassays being more sensitive than LC–MS/MS based assays since immuno-based methods enrich for analytes through antigen–antibody interactions, thus relieving the masking of readout signals by high-abundance proteins [70]. The key to enhancing the sensitivity of LC–MS/MS based assays is to lower ion suppression by reducing sample complexity, for example through LC separation or other separation/enrichment/depletion methods (discussed later in this review). A particularly promising approach is the use of antibody-based enrichment in conjunction with LC–MS/MS, called immunoaffinity enrichment.

## Recent developments in targeted MS-based proteomics workflows and technologies

### Increasing automation

#### Fully automated MS analyzers

To make targeted MS assays more appealing for clinical laboratories, multiple manufacturers have developed fully automated clinical MS analyzers that encompass all necessary steps from sample preparation to result generation. They claim to combine MS’s specificity and sensitivity with the ease of use of immune-based assays. However, so far these systems have not been successfully integrated into the clinical laboratory market. Two previously available systems, the Cascadion SM Clinical Analyzer by Thermo Fisher Scientific and the Topaz analyzer by SCIEX, have been discontinued [71]. In clinical studies, the Cascadion showed good analytical performance and ease of use for quantifying Vitamin D and immunosuppressants [72, 73], but the test menu was never expanded beyond these two applications, which likely contributed to its eventual discontinuation [71]. The Topaz system by SCIEX, in contrast, allowed for integration of additional analytes by the clinical laboratory but was never fully automated [71]. Despite these initial failures to introduce automated MS analyzers to clinical laboratories, Shimadzu and Roche are planning to release their own analyzers in the near future [74]. Both systems are going to offer a bigger test menu than the previously mentioned platforms [75, 76], which should increase their appeal for clinical laboratories. Additionally, Roche announced that their automated MS analyzer will be fully compatible with their Cobas in vitro diagnostics assay platform. This will make it easier to integrate into existing clinical lab workflows and enable, for example, automated follow-up testing of samples positive for drugs of abuse via immunoassays [75]. None of these fully automated MS systems have been applied to protein analysis yet, likely because MS-based analysis of small molecules has a longer history in clinical laboratories. Nonetheless, the integration of protein analysis into these platforms should be feasible.

#### Automated proteomics sample preparation

Sample preparation for MS-based proteomics is time-consuming and, when performed manually, prone to higher imprecision and error rates. In order to improve precision, throughput and processing speed, automated liquid handling workstations have been introduced to proteomics sample preparation (for a recent review see Fu et al. [65]). Automation instrumentation and associated consumables can contribute to increased cost and therefore should be balanced with labor savings through limiting hands-on time. Features such as accurate time-controlled liquid transfer and temperature-controlled incubators with shaking ability reduce hands-on processing time and increase accuracy and reproducibility for large numbers of samples [65]. Automated liquid handlers have already been used to develop clinically validated LC–MS/MS assays for protein/peptide analytes. For example, DeMarco et al. developed an HPLC-MRM assay to quantify wildtype and variant amyloid β in CSF of patients with cognitive complaints suspected of Alzheimer’s disease [77]. Taylor et al. utilized an automated liquid handler in the development of a LC–MS/MS assay to quantify insulin and C-peptide in sera of healthy, insulin resistant, prediabetic and diabetic individuals [78]. Automated liquid handlers are compatible with various proteomics protocols, including direct digestion from dried blood spots or volumetric absorptive microsampling [79, 80], solid phase extraction [81], and immunoaffinity enrichment [58, 65] and have the potential to standardize MS-based proteomics workflows to better meet clinical laboratory standards.

#### Software capabilities for workflow automation

For full integration into the clinical setting, LC–MS/MS systems should be interfaced with Laboratory Information Management Systems (LIMS) and be equipped with software enabling audit trailing and automated data analysis. This improves the efficiency and compliance of laboratory processes and reduces pre- and post-analytical errors. LIMS act as the interface between the HIS (or electronic medical records) and laboratory instrumentation, is used for managing data related to lab test requisitions, patient specimens and patient demographics, and allows for electronic transmission of test order information and lab test results. Key functions of software capabilities include two-way instrument integration (i.e., information is delivered to and from the LC–MS/MS system by the LIMS), automated data analysis, tracking of samples, consumables, reagents, workflows, maintenance, and quality control [82]. As an example, Panorama by LabKey offers software designed specifically for targeted MS-based proteomics. Panorama was conceptualized in 2014 as an online repository for targeted MS/MS data created with Skyline, a popular Windows client application for targeted proteomics method creation and quantitative data analysis [84, 85]. It has since been enhanced with functions for QC monitoring, audit logs, and sample and workflow tracking [86, 87].

### Improving throughput

#### Fast digests

The proteolytic digestion step is one of the most time-consuming steps in targeted proteomics assays. One widespread technique to speed up this process is the use of modified digestion enzymes together with high temperatures (60–70 ℃ as opposed to 37 ℃), which results in digestion times of 30 min to 1 h. Many companies offer enzymes for high-temperature, fast digestion, including Thermo Fisher Scientific (SMART Digest^™^), Sigma Aldrich (SOLu Rapid Digestion), and Promega. Other approaches utilize high pressure [88, 89]immobilized trypsin [25, 90, 91], or on-bead digestion [22] to speed up the proteolytic digestion.

#### MStern

Another process that can limit throughput of targeted proteomics assays is the removal of denaturing agents prior to digestion. Membrane-based approaches like filter-aided sample preparation (FASP) [92, 93] perform proteolysis on membranes with molecular weight filters. First, proteins are bound to the filter, while compounds with a low molecular weight (such as salts and detergents used as denaturation agents) pass through the filter. This is followed by on-filter digestion of the proteins and subsequent elution of the resulting peptides through the filter [2]. FASP increases digestion efficiency and reduces sample loss compared to classical in-solution digestion [93]. The MStern protocol is a high-throughput advancement of FASP, which enables processing in a 96-plate format [94]. As such, it is particularly interesting for clinical applications and has been applied, amongst others, to urine and CSF [94].

#### Evosep one

One recent advancement improving the throughput of LC–MS/MS based proteomics was the introduction of the Evosep One LC system [95]. It utilizes two-dimensional LC, where peptides are pre-separated via a disposable trap column and then transferred to the analytical column for a more in-depth separation [95]. Its setup enables parallel sample loading and analytical separation, which significantly reduces overhead time and enables in-depth proteomic profiling of 60 plasma samples per day [95]. The system has been developed as a more robust and high-throughput alternative to the standard technique for discovery proteomics, nano-flow LC [95], but has potential for applications in targeted proteomics.

### Increasing sensitivity

One of the main avenues to increase the sensitivity of LC–MS/MS assays is to reduce the complexity of the sample before MS analysis, either during sample preparation or via LC separation. Processing methods for the reduction of proteomic sample complexity include, for example, strong cation exchange fractionation [96], high pH fractionation [97], glycopeptide enrichment [44, 98], or extracellular vesicle enrichment [4, 99]. Most of these methods are used in discovery applications, but they are often unattractive for clinical routine analysis since they are labor-intensive, time consuming, and associated with analytical variability [100]. Choosing the right approach for a clinical targeted proteomics assay requires to find a balance between assay sensitivity and turnaround time and many discovery proteomics workflows favor sensitivity over turnaround time and throughput. However, there are several approaches that can achieve the turnaround time and throughput necessary for clinical applications.

#### Immuno-based methods

Immuno-based enrichment and depletion approaches are useful tools to improve the sensitivity of MS-based proteomics assays. One common application is the depletion of high abundant proteins from complex biofluids, such as serum [6], CSF [29], or urine [28] to enhance the detection of low-abundance proteins. However, immunoaffinity depletion can lead to nonselective loss of proteins that are bound to the proteins targeted for depletion [101]. Another application, immunoaffinity enrichment (IAE), greatly enhances the sensitivity of MS-based assays through the enrichment of proteins or peptides of interest [102]. Methods that combine IAE with MS can generally be developed faster and cheaper than traditional immunoassays, since the antibodies used for enrichment don’t have to be as specific due to the use of MS as a specific detector [100]. IAE on the peptide level additionally avoids interferences by endogenous auto-antibodies or anti-reagent antibodies [100] and can expose previously inaccessible epitopes [65]. The combination of IAE on the peptide level with the addition of SIS peptides prior to enrichment is called immune MRM or SISCAPA (Stable Isotope Standards and Capture by Anti-Peptide Antibodies) [65, 103]. SISCAPA has been applied to plasma, serum, and dried blood spots [65] and has been shown to increase sensitivity compared to targeted MS assays without IAE [41]. It is compatible with automated workstations [65, 80, 102] and permits the use of LC gradients as short as 10 min [50] (for comparison, the typical routine clinical LC–MS/MS analysis time for small molecules is 2–5 min [17, 26]).

#### Seer proteograph^™^ assay

The Seer Proteograph^™^ assay was introduced in 2020 as a novel platform for the fractionation of proteomics samples for discovery applications [104]. It utilizes magnetic nanoparticles that attract proteins based on their physicochemical properties to enrich for proteome subsets prior to LC–MS/MS analysis. Over 250 nanoparticle types are available, however a combination of ten nanoparticles has been shown to be able to capture the whole dynamic range of plasma [104]. One recent study detected a record number of over 6000 protein groups in plasma enriched with a Seer Proteograph^™^ Assay kit [105]. The assay platform includes consumables, an automated processing platform, and data analysis tools (so far for untargeted proteomics) which increases the assay’s reproducibility and throughput. However, the protocol still includes manual steps such as for peptide quantification and an overnight drying step [106], requiring a relatively long processing time for routine clinical analysis. Nonetheless, the Seer Proteograph^™^ Assay might have potential for clinical MS-based targeted proteomics assays, providing it can achieve the necessary precision and turnaround time.

#### Multidimensional LC

Another approach to reduce the complexity of proteomic samples is the utilization of multiple LC separations in a row, such as in the Evosep One LC system (see above). This methodology is especially useful for the development of assays for targets without available antibodies [107]. For example, Nie et al. developed a method called Deep Dive SRM (DD-SRM) for the quantification of low-abundance proteins in nondepleted serum without IAE that improved SRM sensitivity by circa 5 orders of magnitude when compared to conventional LC-SRM [107]. They combined a first separation via low pH reversed phase (RP) LC with a second separation via high pH RP LC, followed by traditional RP-LC-MRM [107]. However, multidimensional LC-separation takes time (over 3 h per sample in the case of DD-SRM [107]), making this a low throughput method.

### Ion mobility mass spectrometry

Ion Mobility Spectrometry (IMS) is a widely used analytical technique for separating and detecting ions based on their mobility in a gas phase. Combining IMS with MS/MS adds an extra dimension to the analysis by providing not only the mass-to-charge ratio (m/z) but also the ion's mobility characteristics. This enhances the separation and identification capabilities of both techniques and increases sensitivity while decreasing analysis time [108, 109]. IMS-MS for bottom-up proteomics utilizes two approaches, trapped IMS (TIMS) and high field asymmetric waveform ion mobility spectrometry (FAIMS). In TIMS, precursor ions are trapped and separated according to their shape and charge in an IMS cell before eluting into the mass spectrometer [108]. In one study, TIMS was combined with PRM to quantify the levels of 125 proteins in human plasma over four to six orders of magnitude using a 40 min LC gradient [109]. Quantification results correlated well (R2 = 0.97, slope 0.99) with a traditional MRM method on a quadrupole instrument and allowed absolute protein quantitation down to 1.13 fmol [109]. FAIMS separates gas-phase ions by their behavior in strong and weak electric fields and improves the dynamic range and sensitivity of MS assays by removing interfering ion species and selecting peptide charge states optimal for identification by tandem MS [110]. It has been applied to the quantification of a peptide drug candidate in rat plasma [111] and to the PRM analysis of ten peptides representing oncology drug targets and biomarkers in FFPE tissue [112]. FAIMS-PRM reduced background signals compared to PRM which increased assay sensitivity, enabling quantitation of basal HER2 expression in breast cancer samples classified as HER2 negative by immunohistochemistry [112]. However, IMS-compatible MS/MS instruments are generally cost-prohibitive (500,000–1,000,000 USD).

### MS-based targeted proteomics for clinical cancer biomarker analysis—future directions

In summary, targeted MS-based proteomics is a specific technique for the precise quantification of cancer markers in biofluids with great potential for clinical applications in oncology. Recent developments have improved the throughput, reproducibility, and sensitivity of MS-based targeted proteomics, making the technique more accessible and attractive for clinical implementation. One remaining hurdle for the wide-spread implementation of targeted MS-based proteomics assays in the clinic is the high technical proficiency required for LC–MS/MS handling. To date, most clinical LC–MS/MS assays are laboratory developed tests where the clinical laboratory is responsible for method development, validation, and post-implementation monitoring [33] which makes LC–MS/MS based assays less appealing to smaller laboratories.

However, MS-based targeted proteomics assays show higher reproducibility than immunoassays [59], which suffer from low long-term and inter-assay/inter-lab reproducibility [39, 60, 113]. Therefore, implementing more LC–MS/MS-based assays into clinical laboratories is expected to lead to higher concordance between results generated over a long period of time and/or by different laboratories. This will help to standardize patient care cross healthcare systems and enable the consistent long-term monitoring of health conditions, the adoption of decision cut-offs, and the comparison of clinical studies [113]. Additionally, targeted proteomics assays have a lot of promise in the developing fields of multi-biomarker panels and precision medicine due to their multiplexing capability, sample efficiency, and specificity. Relying on single or few biomarkers is limiting, particularly in diseases with diagnostic gaps, in situations with complex differential diagnoses and in patients with multiple comorbidities [114]. Further implementation of multiplexed MS-based proteomics assays to measure biomarker panels will lead to clinical tests with higher diagnostic and predictive value. Additionally, precision medicine approaches that tailor medical care and treatment decisions to patient-specific characteristics such as proteome profiles [115] will greatly facilitate patient stratification, treatment selection, and treatment monitoring [51].

## Data Availability

Not applicable.

## Abbreviations

AUC: Area under the curve
CA125: Cancer antigen 125
CLSI: Clinical & Laboratory Standards Institute
CSF: Cerebrospinal fluid
ELISA: Enzyme-linked immunosorbent assay
ESI: Electrospray ionization
FAIMS: High field asymmetric waveform ion mobility spectrometry
FASP: Filter-aided sample preparation
FDA: Food and Drug Administration
HIS: Health Information System
HPLC: High pressure liquid chromatography
IAE: Immunoaffinity enrichment
IgG: Immunoglobulin G
IMS: Ion mobility spectrometry
LC: Liquid chromatography
LDT: Laboratory developed test
LIMS: Laboratory information management system
MRM: Multiple reaction monitoring
MS: Mass spectrometry
MS/MS: Tandem mass spectrometry
PRM: Parallel reaction monitoring
PSA: Prostate-specific antigen
PTHrp: Parathyroid hormone–related peptide
RP: Reversed phase
SIS: Stable isotope standard
SISCAPA: Stable Isotope Standards and Capture by Anti-Peptide Antibodies
SOP: Standard operating procedure
SRM: Single reaction monitoring
SSA: System suitability assessment
TIMS: Trapped ion mobility spectrometry
